# Diagnosing attention-deficit hyperactivity disorder (ADHD) using artificial intelligence: a clinical study in the UK

**DOI:** 10.3389/fpsyt.2023.1164433

**Published:** 2023-06-09

**Authors:** Tianhua Chen, Ilias Tachmazidis, Sotiris Batsakis, Marios Adamou, Emmanuel Papadakis, Grigoris Antoniou

**Affiliations:** ^1^Department of Computer Science, University of Huddersfield, Huddersfield, United Kingdom; ^2^School of Production Engineering and Management, Technical University of Crete, Chania, Greece; ^3^South West Yorkshire Partnership National Health Service (NHS) Foundation Trust, Wakefield, United Kingdom; ^4^L3S Research Center, Leibniz University Hannover, Hannover, Germany

**Keywords:** attention-deficit hyperactivity disorder (ADHD), diagnostic system, artificial intelligence, machine learning, explainable AI, mental health

## Abstract

Attention-deficit hyperactivity disorder (ADHD) is a neurodevelopmental disorder affecting a large percentage of the adult population. A series of ongoing efforts has led to the development of a hybrid AI algorithm (a combination of a machine learning model and a knowledge-based model) for assisting adult ADHD diagnosis, and its clinical trial currently operating in the largest National Health Service (NHS) for adults with ADHD in the UK. Most recently, more data was made available that has lead to a total collection of 501 anonymized records as of 2022 July. This prompted the ongoing research to carefully examine the model by retraining and optimizing the machine learning algorithm in order to update the model with better generalization capability. Based on the large data collection so far, this paper also pilots a study to examine the effectiveness of variables other than the Diagnostic Interview for ADHD in adults (DIVA) assessment, which adds considerable cost in the screenining process as it relies on specially trained senior clinicians. Results reported in this paper demonstrate that the newly trained machine learning model reaches an accuracy of 75.03% when all features are used; the hybrid model obtains an accuracy of 93.61%. Exceeding what clinical experts expected in the absence of DIVA, achieving an accuracy of 65.27% using a rule-based machine learning model alone encourages the development of a cost effective model in the future.

## 1. Introduction

Attention-deficit hyperactivity disorder (ADHD) is a neurodevelopmental disorder characterized by symptoms of inattention, hyperactivity, and/or impulsivity that causes significant impairment across domains. People with ADHD also exhibit deficits in executive functions, behavior and emotion regulation and motivation (1). Global demand for ADHD diagnostic assessment is rapidly growing due to increased awareness of the condition and other possible factors like impact of the pandemic (2). Within the UK where the conducted research is trialed in clinical practice, ADHD affects about 3–5% of children and 2% of adults (3).

In case of ADHD diagnosis the modes of intervention according to the National Collaborating Centre for Mental Health, UK, are both pharmacological and psychological (4). The first line treatment for adult ADHD is psychostimulants (5) and medication is safe and effective, with 70% of patients reported improvement compared to 7% of controls (5, 6).

Delayed diagnosis and treatment for ADHD can be harmful to people and may cause broader mental health conditions, relationship and employment problems, criminal activities, and substance misuse. Specifically the adverse effects of untreated ADHD are well-documented with negative effects on academic outcomes (7), social functioning (8), employment (9) but also life itself leading to increased mortality (10).

For the UK, the National Institute for Health and Clinical Excellence (NICE) suggested in 2008 that the standard benchmark rate for referral to a Service in adults is 25 per 100,000 per year. The largest challenge at the moment for the adult population, bearing in mind the relative recency of acceptance amongst the professional community that ADHD can persist into adulthood (11), is the dearth of clinicians appropriately trained and confidence to place the diagnosis. Such bottleneck prevents patients receiving appropriate treatments and hence contributes to the morbidity of the adult ADHD.

The increased demand for assessments combined with the shortage of adequate healthcare capacity led to excessively long waiting lists, with an average waiting time up to 3 years. This puts a significant economic burden on the NHS, social services and the state overall. The total yearly costs to the individual and state combined were recently estimated to be €17,769 per person, per year (12) thus suggesting there is strong impetus for action.

In order to handle these challenges and coupled with the fact that Artificial Intelligence (AI) is enjoying an increasing number of successes in medical applications (13–16), an AI system, called NeuroIntel, was developed. For this work, clinical information collected from an NHS adult ADHD Service, which delivers a clinical pathway compliant with NICE recommendations (i.e., the gold standard), was used for creating a decision support tool that can first automate the process of making a diagnosis and second prioritize the ADHD cases based on levels of complexity. This prioritization serves to select the patients which would require a more in-depth clinical assessment. The clinical data collected were in the form of screening questionnaires and validated clinical diagnostic interviews, which are routinely collected as part of a clinical diagnostic assessment.

Applying machine learning for ADHD diagnosis (17) is a recent approach for dealing with this issue. Being commonly used in medical settings where the demand of interpretability is generally considered high, knowledge-based systems aim to represent knowledge explicitly via tools such as production or if-then rules, which allow such a system to reason about how it reaches a conclusion and to provide explanation of its reasoning to the user (18). In order to combine the strengths of machine learning-based approaches with the interpetability of knowledge based systems these approaches were combined in a hybrid setting (19), such that patterns extracted by machine learning and expertise directly given by clinicians can be unified in a single framework that best maximizes both approaches.

A series of efforts (17, 19) have been invested that has lead to the deployment of existing hybrid systems in the Adult ADHD Service of South-West Yorkshire Partnership NHS Foundation Trust (SWYPFT). The initial exploration (17) made use of data sources including both structured patient information as well as unstructured textual medical notes, but only on the basis of available electronic records from 69 patients only, with decision tree learning algorithm identified as an optimum choice to construct the diagnostic model, owing to its superior performance and interpretability. Another outcome of the underlying study also suggested the inclusion of features extracted from medical notes did not necessarily enhance the predictive capability, but might ran the risk of overfitting the models. A hybrid model (19) was subsequently proposed, which aims to not only utilize patterns learned by machine learning, but also incorporate expertise from senior clinicians, with results showing great promise of the technology, as it can accurately identify clear-cut cases where a decision can be safely made and can be verified by a less senior clinician, while referring the more complex cases for further assessment by a senior clinical specialist. With an ongoing trial operating in the largest NHS Service for adults with ADHD in the UK, the ongoing data collection has lead to the accumulation of 285 total patient records, with the evaluations and results as a retrospective study currently under review.

Most recently, more data was made available that has lead to a total collection of 501 anonymized records by 2022 July. This prompted the ongoing research to carefully examine the model with the large data collection so far, as reported in this paper, with the following major contributions:

1. In this paper, we aggregated all cases collected so far into one data set, followed by retraining and optimizing the machine learning algorithm in order to update the model with better generalization capability.
2. Efforts so far has made full use of all available variables in all the models, including the Diagnostic Interview for ADHD in adults (DIVA) assessment. While relevant to the assessment process, DIVA adds considerable burden as it relies on specially trained clinicians. Given the huge demand for diagnosis, both primary and secondary healthcare providers have been seeking for screenings without the possible use of DIVA. Such clinical demand warrants a test on the effectiveness of predictors other than DIVA that is also reported in this research.

The results reported in this paper demonstrate that the newly trained machine learning model reaches an accuracy of 75.03% when all features (including DIVA) are used; the hybrid model combining the new ML model with the knowledge model from (19) obtains an accuracy of 93.61%. When DIVA attributes are disregarded, the best performing machine learning model reaches an accuracy of 65.27%.

The remainder of this paper is organized as follows. Section 2 describes the data used as well as the data analysis framework. Section 3 presents the results of applying machine learning to all available data, as well as to partial data omitting attributes originating from the DIVA. Section 4 analyses and discusses the results, and Section 5 concludes the paper with a summary of the contributions and an outlook on future research.

## 2. Materials and methods

### 2.1. Data collection

For this project, the need for ethics approval was waived by South West Yorkshire Partnership Foundation Trust (SWYPFT) Research and Development Department as data were gathered retrospectively. Data was gathered as part of the clinical operations of the Service and was classed as a service improvement activity. The Caldicott Guardian at SWYPFT endorsed access to data following Caldicott Principles presented at: https://www.highspeedtraining.co.uk/hub/7-caldicott-principles/. Data was gathered from electronic records and patients accessing the Service are routinely informed that their data can be used for research purposes and can opt out if they wish.

The patient's data are provided by an NHS specialist mental health provider (South West Yorkshire Partnership NHS Foundation Trust-SWYPFT). In this study, we have included all cases from the time period it covers, without excluding any patients. For each case, we considered all clinical data routinely collected by the NHS service, following NICE guidelines, ahead of an appointment with a specialist clinician. The approach is to identify not only symptoms of ADHD but also consider comorbid conditions which could also present as ADHD before a diagnosis is made. This is consistent with what the DSM-5 criteria requires which in criterion E requires the clinician to make a judgement that the comorbid conditions do not better explain the presentation.

**Figure 1:**
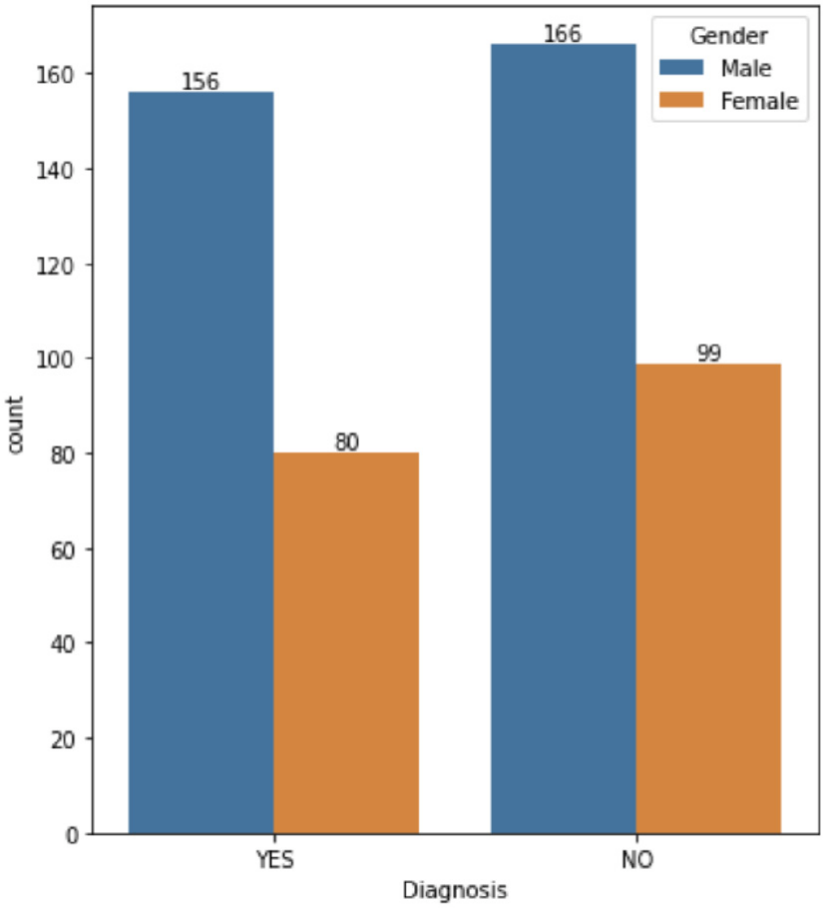
ADHD case vs. gender.

**Figure 2:**
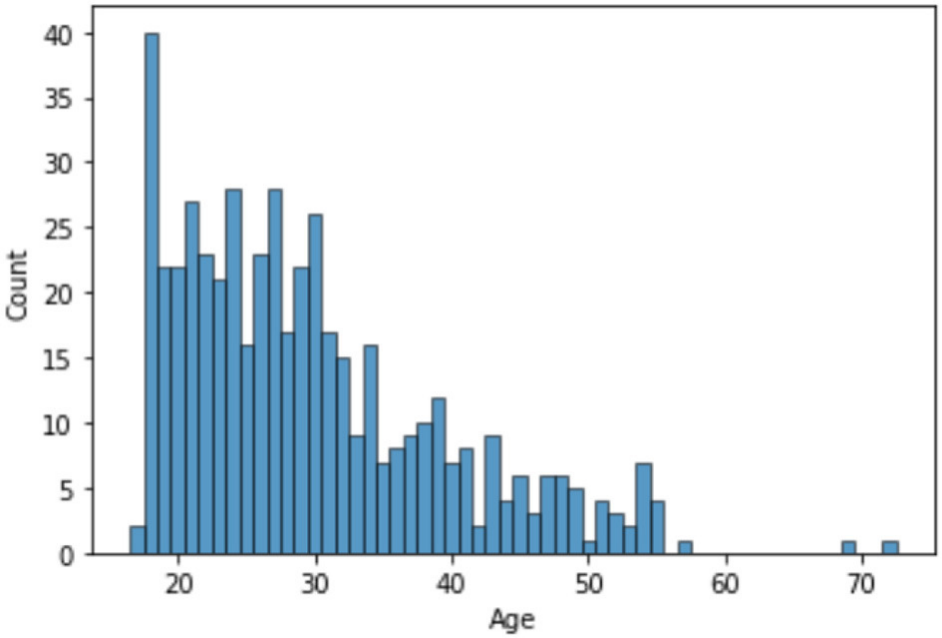
Age distribution.

**Figure 3:**
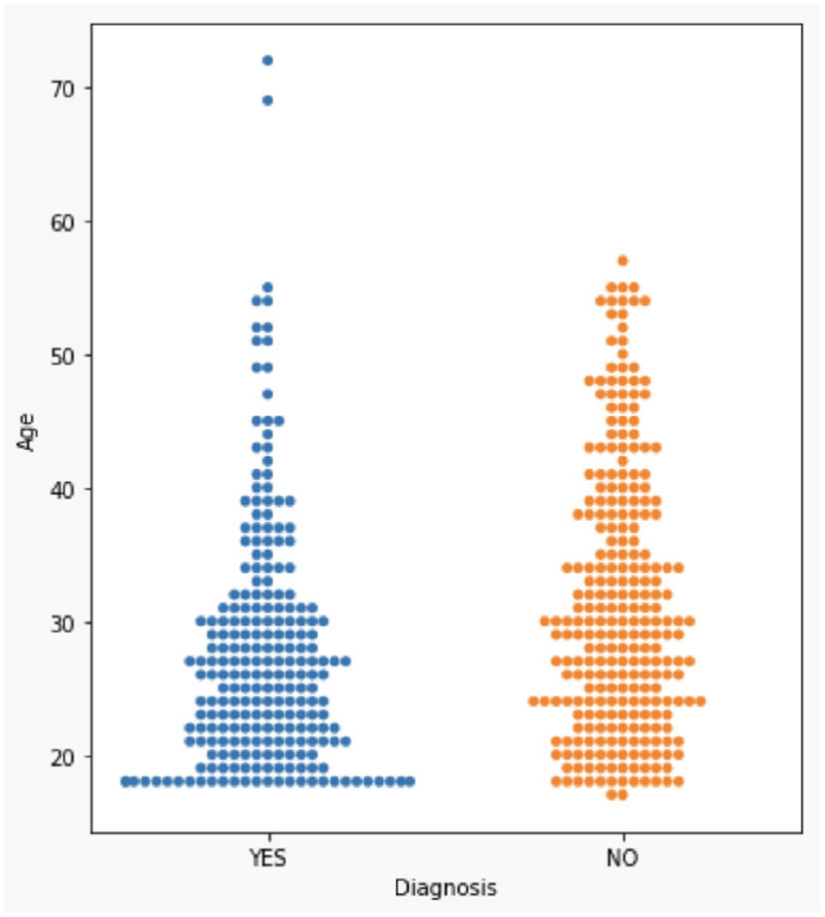
ADHD case vs. age.

The dataset consists of 501 anonymized assessments for ADHD patients in the period between 2019 and 2022 July. The dataset contains demographic information about these patients in addition to self-reported screening questionnaires and clinical interview results. A total of 66 independent attributes are included into the dataset for each case with the last column of the dataset being the diagnostic outcome to predict. With 236 positive cases and 265 negative cases, the distribution of class labels is relatively balanced; whereas male subjects (322) are nearly twice that of female (179), in Figure 1, where it also shows the gender distribution for each of the diagnoses. In terms of the age distribution, Figure 2 suggests the age group between 20 and 30 has the most patients, with the youngest patient being 17 while the most senior being 72. The swarm plot shown in Figure 3, further suggests that, in general, ages of both positive and negative cases span from just below 20 to around 55, except for a couple of positive cases where age is around 70.

The data for each patient includes a patient identifier and the patient's gender and age. This is followed by the results of the Mood Disorder Questionnaire (MDQ) (20), the HELPS brain injury screening tool (21), the Drug Abuse Screening Test (DAST-10) (22), the GAD-7 test results measuring Generalized Anxiety (23), the Patient Health Questionnaire (PHQ-9) which measures the severity of depression (24), the Iowa Personality Disorder Screen (IOWA) (25), the Alcohol Use Disorders Identification Test (AUDIT) (26), the Conner's ADHD Rating Scales (27) and the Diagnostic Interview for ADHD in adults (DIVA) (28) results.

### 2.2. Diagnostic process

The diagnostic process follows best practice approach recommended by the Royal College of Psychiatrists in the UK ADHD in adults: Good practice guidance (CR235) (29). The approach recommends a list of validated screening and diagnostic tools as well as a formal exploration of comorbidity. The inputs we used to construct the diagnostic tool capture these recommendations by capturing all components of the diagnostic process by using screening tools for ADHD and mental health, validated diagnostic tools for ADHD and a process for considering comorbidity with other conditions. As such the clinical diagnostic process has three steps: first, collection of information using screening tools (which also include input by carer); second, administration of a validated diagnostic tool (DIVA); third, full psychiatric history to validated findings and identify comorbidities which could explain the presentation.

**Figure 4:**
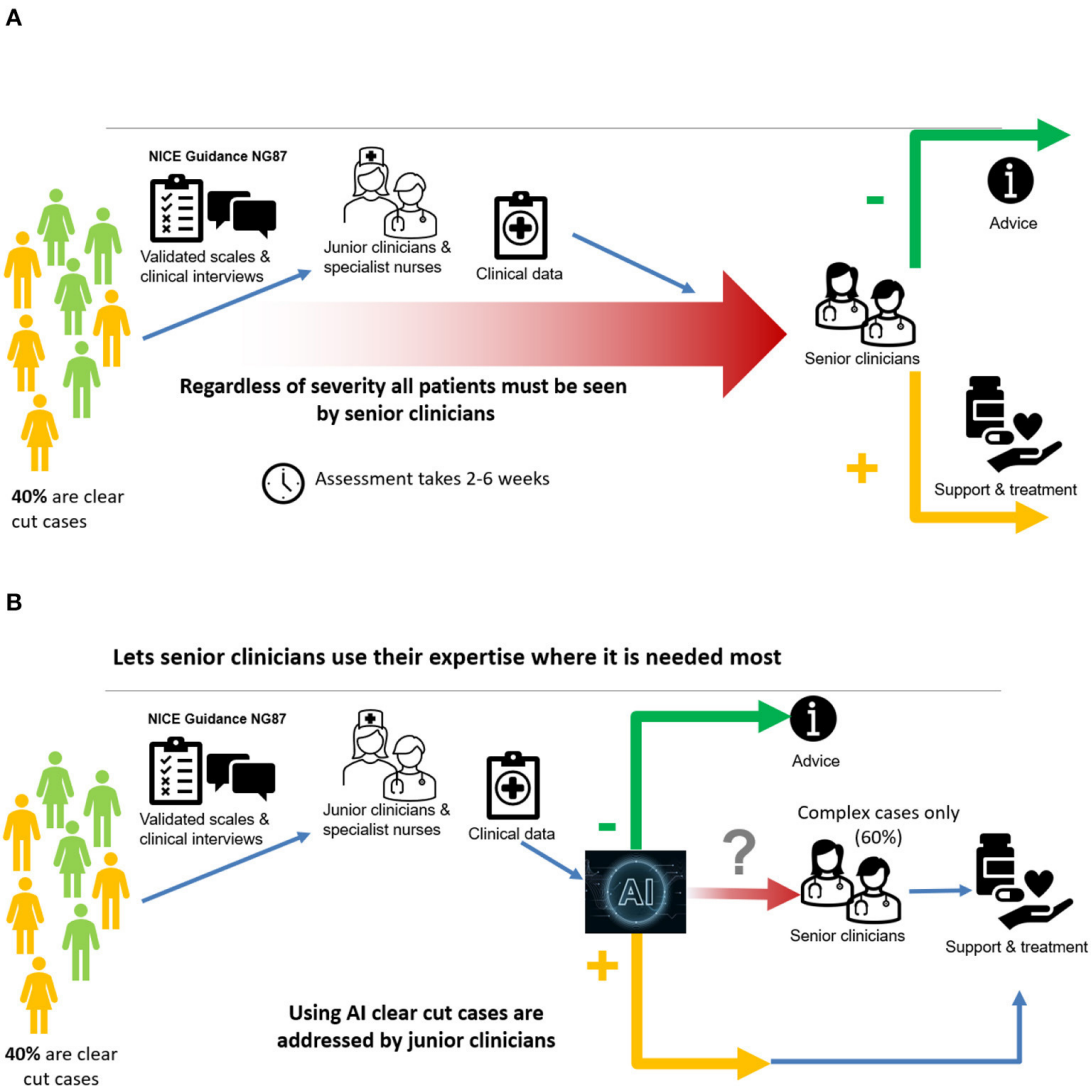
**(A)** A major bottleneck in current pathway; **(B)** AI overcomes the major bottleneck.

A schematic description of the current approach is shown in Figure 4A, while the use of the AI tool is illustrated in Figure 4B.

### 2.3. Framework architecture

Based on the analysis of the dataset, a diagnostic system for adult ADHD diagnosis has been developed consisting of two parts: (a) the machine learning (ML) model and (b) the knowledge representation-based (KR) model. The diagnostic outcomes of these two models are also combined producing the hybrid model. The system consisting of the ML, KR, and hybrid models has been deployed and used for assisting clinicians for ADHD diagnosis, offering an intuitive Web-based interface. In the following, the components of the system are presented.

#### 2.3.1. Machine learning model

In order to develop a prediction model using machine learning, a number of mainstream algorithms were evaluated, with the evaluation results presented in detail in Section 3. The fact that a decision tree model is adopted, is due partially to the robust performance it offers in comparison with alternative popular machine learning models, but also to the interpretability it offers to represent the learning model through a set of IF-THEN rules (18). Such rules are highly recommended by healthcare professionals who not only are enabled to interrogate inference made by a machine learning model, but also makes it possible to integrate human knowledge for the ultimate generation of a hybrid system that incorporates both patterns extracted by a learning system and expertise from clinicians, as reported in our recent work (19).

The input to the model is a set of numerical values aggregating the full set of features of the initial dataset. Apart from the Age attribute, a number of psychological measures are used, i.e., PHQ9: Severity of self reported depression (numerical, having values between 0 and 27); GAD: Severity of self reported anxiety (values 0–21); MDQ: Self reported symptoms of bipolar disorder (Boolean value); AUDIT: Harmful alcohol consumption scale (values 0–40); DAST10: Drugs use score in the last 12 months (values 0–10); HELPS: Exposure to brain injury during lifetime (Boolean value); IOWA: Personality disorders evaluation (values 0 to 11); CAARS: CAARS ADHD TT1 score (values 1–100); DIVA Child IA: Attention deficit during childhood score (values 0–9); DIVA Child HI: Hyperactivity/impulsivity during childhood score (values 0–9); DIVA Adult IA: Attention deficit during adulthood score (values 0–9); DIVA Adult HI: Hyperactivity/impulsivity during adulthood score (values 0–9). The machine learning model receives the above mentioned input and produces as output of the corresponding rules a Boolean diagnostic outcome (“Yes” or “No”).

#### 2.3.2. Knowledge based model

The knowledge model (19) for ADHD diagnosis encodes the empirical knowledge of an international expert in adult ADHD. This expert knowledge was extracted through interviews in order to encode the deep understanding of various tests and questionnaires that are routinely conducted by SWYPFT Research and Development Department (see Section 2.1). The meaning of each source of data was explored during the interviews and encoded in a machine-readable format in the form of if-then rules. Once rules were defined, their priority needed to be further specified in order to emulate the rationale of a clinical expert.

The knowledge model relies on DIVA scores, with low DIVA scores indicating that ADHD should not be inferred, while high DIVA scores indicate that ADHD diagnosis is more probable. A holistic approach is required, were patients affected by substance abuse, personality disorder, alcohol use, bipolar disorder, anxiety, depression and brain injury, might exhibit overlapping symptoms with ADHD. Thresholds are set to quantify abstract notions such as low or high DIVA scores, with rules prioritized in order to recreate the decision making process of a clinical expert. The knowledge model allows three possible outcomes, namely “Yes” (positive diagnosis), “No” (negative diagnosis), or “Expert” (the case should be referred to a clinician). For further details about the knowledge model, readers are referred to (19).

#### 2.3.3. Hybrid model

The hybrid model (19) combines the results of the knowledge model and the machine learning model. Notice that the hybrid model requires the use of all available data (as opposed to the alternative machine learning model without DIVAs). A key difference between the two models above is that the machine learning model provides yes/no answers, while the knowledge model provides yes/no/expert answers. The hybrid model provides yes/no answers when both machine learning and knowledge model are in agreement. When the two models are in disagreement, patients are referred to a medical expert.

The main advantage of the hybrid model is that its yes/no answers are endorsed by both machine learning and knowledge model. Moreover, the machine learning model provides its recommendation to clinical specialists toward a particular outcome, even for patients that are referred to an expert (by the hybrid model). It is worth noting that referring patients to medical experts is a valid and desirable outcome since AI is aimed at streamlining clear-cut cases as well as identifying complicated cases that require expert's analysis. For further details about the hybrid model, readers are referred to (19).

**Figure 5:**
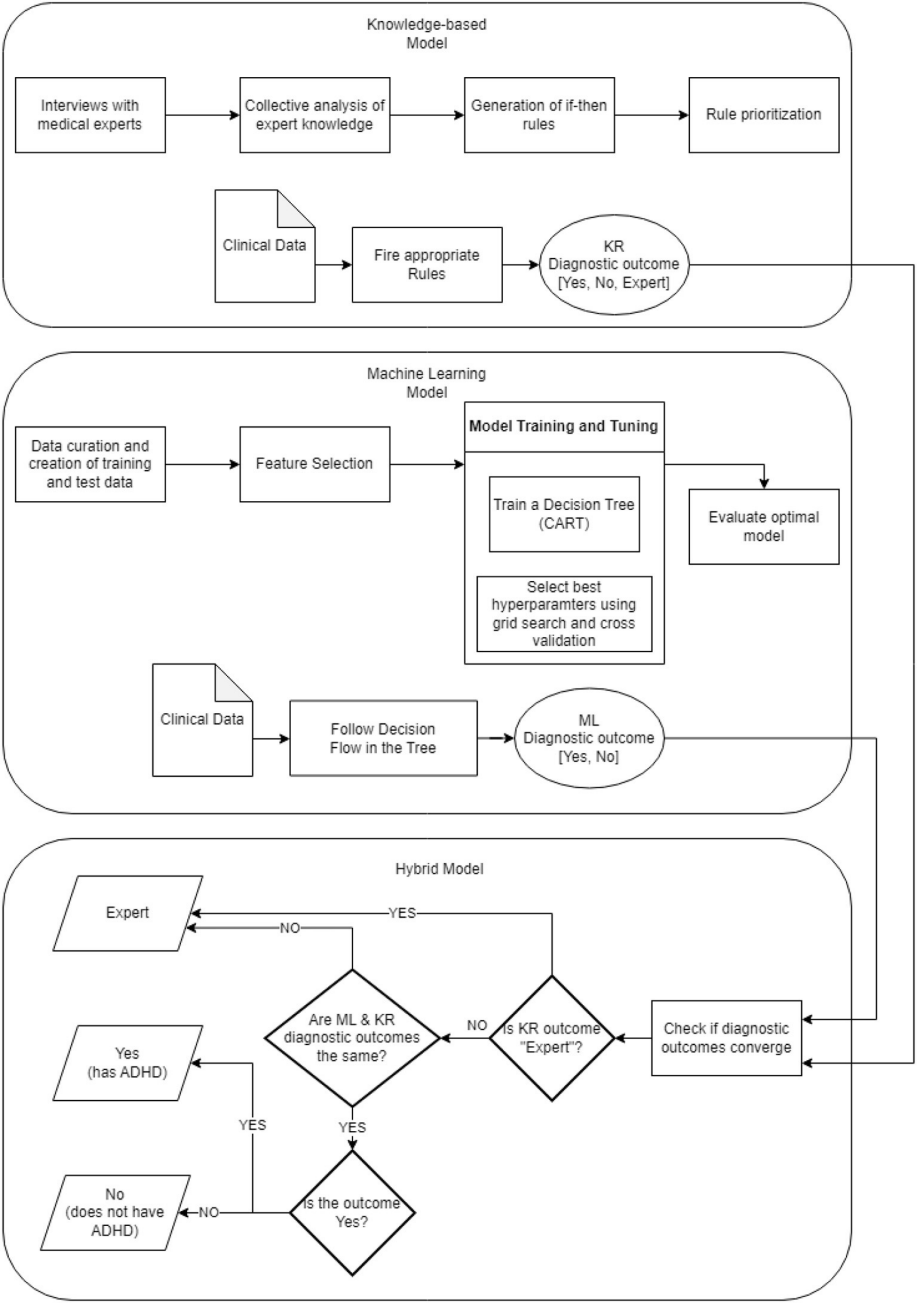
ADHD diagnostic system framework.

Figure 5 depicts the overall framework of all the components mentioned above, which build the proposed diagnostic system. The visual is organized in three parts reflecting the corresponding models; each model is detailed in terms of its construction pipeline along with a brief overview of its functionality. To this end, a new assessment is processed by creating two diagnostic outcomes based on the rules set and the decision tree underlying the knowledge-based and machine learning component, respectively. Both outcomes, then, are combined by the hybrid model where the convergence of the diagnostic result is assessed, which eventually results into the final diagnostic recommendation.

## 3. Results and discussion on machine learning models

Correlation analysis is a statistical method used to examine the relationship between two variables, which allows determining whether there is a relationship and the strength between the two variables. To understand better such potential relationships before building the predictive models using machine learning algorithms for diagnosing ADHD, a correlation analysis is therefore conducted for each of the independent variables against the “Diagnosis” dependent variable.

**Figure 6:**
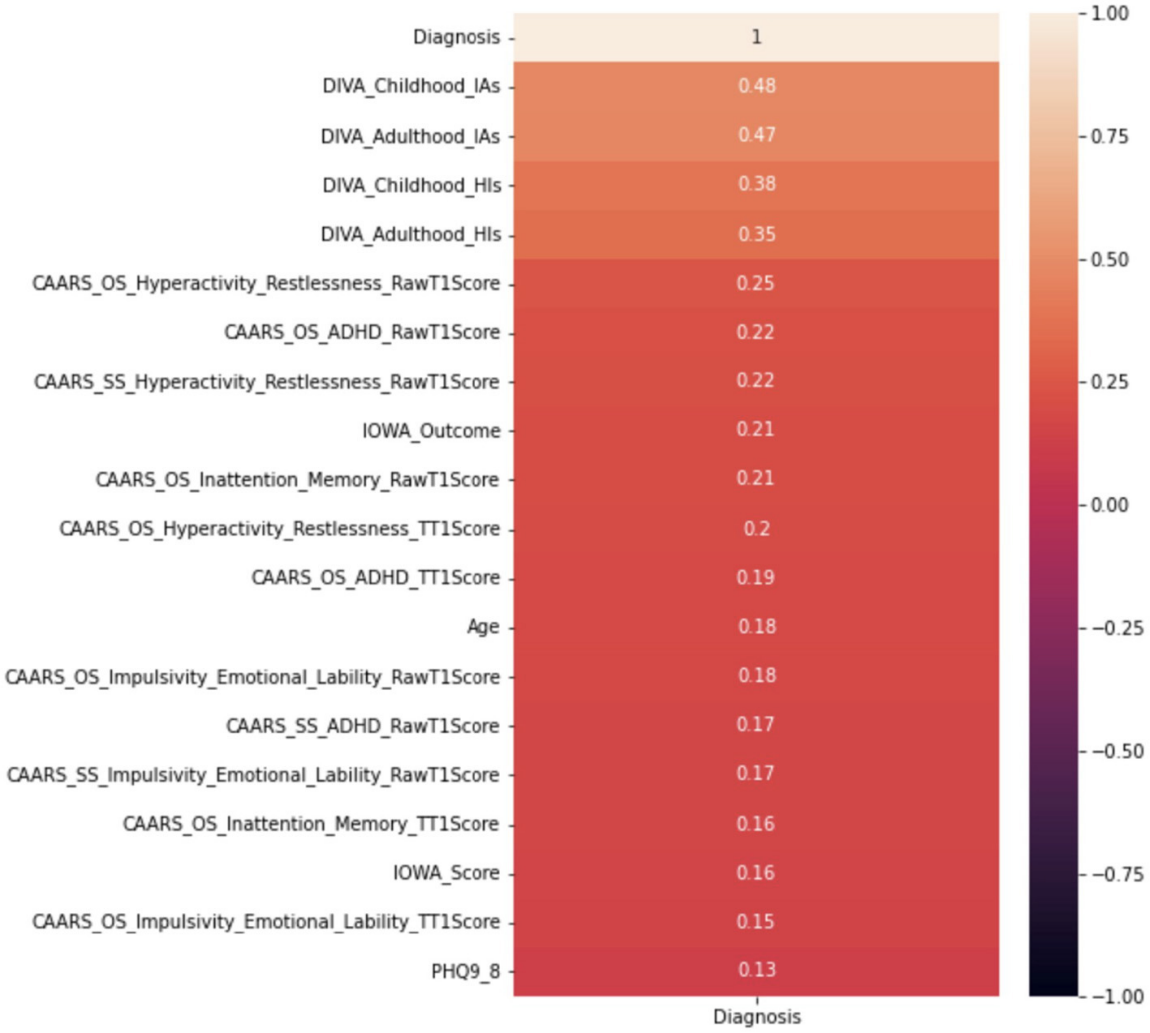
Correlation analysis of top 20 attributes including DIVAs.

This paper adopts the popular Pearson correlation with the value ranging between +1 and −1, where a value of +1 is a total positive linear correlation; 0 is no linear correlation; and −1 is a total negative linear correlation. In order to measure the strength of the correlation, absolute values of the actual correlation are used. Owing to the space limit, Figure 6 demonstrates the strength of the correlation of the top 20 independent variables. The DIVA attention deficit for both childhood and adulthood presents the strongest correlation with the diagnosis, followed by the DIVA hyperactivity/impulsivity for both childhood and adulthood. This is followed by a weaker set of attributes around CAARS ADHD TT1 score, the IOWA personality disorders evaluation and age. It is important to note that correlation analysis does not necessarily imply causation, though they can be useful observations for following analysis.

While the knowledge model, which directly comes from clinical expertise, remains relatively stable; the machine learning model, which is data-driven in nature, is subject to re-train, given the significant recent intake of data from 216 new patients, making the total data entries 501. In particular, the decision tree algorithm, which has been used consistently, for its effectiveness in diagnostic accuracy as well as the inference interpretable by clinical professionals, remains our first choice among alternative machine learning models, which is consolidated by our successes so far as also highly recommended by the clinicians (17, 19).

### 3.1. ML results using all available data

Decision tree learning has been one of the most influential machine learning and data mining algorithms (30), where it recursively selects the most informative attribute that returns best homogeneous sets of the underlying data instances, until all attributes have been considered or the addition of any remaining attribute does not improve its discriminative power. While more details on the induction of a decision tree can be found in (30), the specific Classification and Regression Tree (CART) is utilized for experimenting with the newly collected ADHD data. Using Google Colab, the implementations of the algorithm comes from Scikit-learn (31), which is a free software machine learning library for the Python programming language.

In identifying the optimal decision tree model that best fits the underlying data, a hyper-parameter search is conducted through a grid search to examine a number of hyper-parameter that might affect model construction, so that multiple instances of CART models can be trained and assessed on the same dataset but initialized with different hyperparameters. In particular, “min samples split” was tested with values 2, 4, 6, 8, 10; this parameter specifies the minimum number of data samples required to create an internal decision node, which eventually protects the model from over-fitting. “Max features” was tested on “sqrt” and “log2”, which defines the number of features required to make a split decision. “Min samples leaf”, similar to “min samples split” was set to [1, 8], this parameter sets a threshold of minimum observations for the creation of final decision nodes (leaf nodes). “Max depth” was tested on range [2,6], which is mainly used for preventing overfitting by controlling the size of the final decision tree. Another test took place in the choice of splitting criteria, which is either “gini” and “entropy”—both quantify the level of impurity and disorder and is used to directly guide the selection of a particular attribute to split the tree.

To ensure that the generated model is not overfitting the data with a more fair estimation of the model's generalization error, the k-fold cross-validation is used whereby a model is given a dataset of known data on which training is run and an independent dataset of unknown data against which the model is tested. Specifically, for k-fold cross-validation, the original dataset is randomly partitioned into k equal sized subsamples. Of the k subsamples, a single subsample is retained as the validation data for testing the model, and the remaining k-1 subsamples are used as training data. The cross-validation process is then repeated k times, with a different subsample being used as the validation data each time. The performance measure is calculated by averaging the performance across all k iterations. In this research, the value of *k* is set to 10 for conventional purposes (32).

In terms of the specific metric to examine the performance, results are reported using several metrics, including:

- Accuracy (Acc), in the percentage of correct predictions, i.e., the resultant model predicts positive in case the patient to be diagnosed is with ADHD and negative in case the patient is without ADHD. A perfect classification model would always make correct predictions, resulting in 100% accuracy. Given a model trained on training data, the train accuracy reports the performance on the training data; while the test accuracy is the performance when the trained model is validated on test data that model has never seen before.
- Balanced accuracy, is defined as the average of the sensitivity and specificity of the model, where sensitivity is the proportion of positive cases that are correctly identified by the model, while specificity is the proportion of negative cases that are correctly identified by the model.
- Precision measures the proportion of positive predictions that were actually correct, defined as the number of true positive predictions made by the model divided by the total number of positive predictions made by the model.
- Recall measures the proportion of actual positive cases that were correctly identified by the model, defined as the number of true positive predictions made by the model divided by the total number of actual positive cases in the dataset.
- F1-score is used to balance precision and recall as a measure of a model's overall accuracy, defined as the harmonic mean of the model's precision and recall.
- Auc, the Area Under the Receiver Operating Characteristic (ROC) curve, is the curve of sensitivity (a.k. a. true positive rate), plotted against 1-specificity (a.k.a. false positive rate), which is independent of the prior class distribution, i.e., percentages of positive and negative samples. A perfect classification would produce AUC = 1, while random guessing would produce a 0.5 AUC.

Best result of the CART model after the grid search is then reported in Table 1. However, despite the decision tree being the first choice, it's also critical to evaluate learning algorithms of alternative common choices to give a comprehensive view of the general performance landscape. A multitude of mainstream machine learning algorithms (30) was selected including:

- Logistic Regression, a generalized linear model that uses a logistic function to model the probability of a positive/negative diagnosis given the underlying variables.
- Linear Discriminant Analysis, similar to logistic regression, finds the linear combination of features that maximally separates the different classes, but with additional assumptions on data that logistic regression does not make.
- Multiple Layer Perceptron, widely applied in numerous practical applications, is a type of artificial neural network that consists of multiple layers of interconnected nodes, with each layer fully connected to the next.
- K-nearest Neighbor or KNN is the classical instance-based learning approach, where an instance is classified by a majority vote of its neighbors. It works by assigning an instance to the class most common among its *k* nearest neighbors.
- Support Vector Machine is a sequential optimization algorithm for building support vector machines (which form another type of most powerful learning classifiers), with both linear and Radial basis function (RBF) kernel adopted as kernel function.
- Gaussian Naive Bayes, is based on the Bayes Theorem by using the probability distribution of each feature to make predictions about the diagnosis of a new patient, assuming the probability distribution of each feature follows a Gaussian distribution.
- Random Forest is a very powerful ensemble machine learning method, made up of a collection of decision trees, which are trained on different subsets of the data and then combined to make predictions. The final predictions are made by averaging the predictions of all the individual trees in the forest.
- Extreme Gradient Boosting is another mighty ensemble machine learning method that involves training a sequence of weak decision trees models, and then combining their predictions to form a stronger model.

**Table 1:** Results using all available attributes.

**Data set**	**Train acc**	**Test acc**	**Balanced acc**	**Precision**	**Recall**	**F1**	**Auc**
Classification and Regression Tree	75.05	**75.03**	**75.74**	0.69	**0.88**	**0.77**	0.78
Logistic Regression	75.74	66.85	66.79	0.65	0.66	0.65	0.75
Linear Discriminant Analysis	77.93	64.45	64.44	0.62	0.64	0.63	0.70
Artificial Neural Networks	76.00	66.25	68.84	0.65	0.72	0.66	0.75
K Nearest Neighbor	76.36	60.27	60.04	0.58	0.57	0.57	0.62
Support Vector Machine (RBF)	65.31	62.27	62.09	0.60	0.60	0.60	0.67
Support Vector Machine (Linear)	79.53	64.85	64.83	0.62	0.65	0.63	0.70
Gaussian Naïve Bayes	67.31	66.07	67.06	0.60	0.84	0.70	0.71
Random Forest	**100.00**	74.44	73.34	0.72	0.75	0.71	**0.80**
Extreme Gradient Boosting	**100.00**	72.84	72.84	0.71	0.73	**0.72**	**0.80**
Averaged	79.80	66.48	66.70	0.64	0.68	0.65	0.72

Experiments were conducted using the scikit-learn open source machine learning library that integrates the implementation of all aforementioned ML approaches with default settings unless otherwise explicitly specified. As there also exist missing values in the collected data, all missing values were replaced using simple imputation, i.e., the mean value for numerical data, and the mode for categorical data, though more advanced interpolation technique (33) may be considered in future work.

Table 1 summarizes results of the ten machine learning models across the seven aforementioned performance metrics; the most important observations are highlighted in bold. On the basis of 10-fold cross validation, attention is first drawn to the train and test accuracy, whereby Random Forest and Extreme Gradient Boosting achieves the best possible train accuracy of 100%, which clearly suggest overfitting, with serious gap between train and test accuracies. The remaining model generally achieves 70+% train accuracies with 60+% test accuracies, indicating some slight overfitting. Despite performances of alternative models might be further improved, results based on default parameter settings are generally considered in experimental practice as comparison. In general, testing results all exceed 60%, clearly beating the random guess of 52.9%, which is calculated based on the original distribution of 236 positive and 265 negative cases—these demonstrates the validity of using machine learning models to support decision making of a complex task in clinical practice such as ADHD diagnosis. Among those, the CART decision tree has achieved the test accuracy only very slightly higher train result, suggesting a robustly fitted model when it's trained. Furthermore, its test and balanced accuracy, as highlighted in bold, achieves the best results, clearly beating most competitors by a large margin. In terms of precision and recall, while the precision isn't the best among all, this can be mitigated by the knowledge model and further examination by clinicians, the significantly high recall suggests it only misses a few positive cases that should have been attended to. Whereas the F1, which is an average of the precision and recall, as well as the Auc score, still suggests that CART is among the top accurate models. Overall, with the recent significant collection of new patient data, machine learning is able to enhance the diagnostic accuracy for ADHD, and decision tree is still a robust choice from performance perspective.

**Figure 7:**
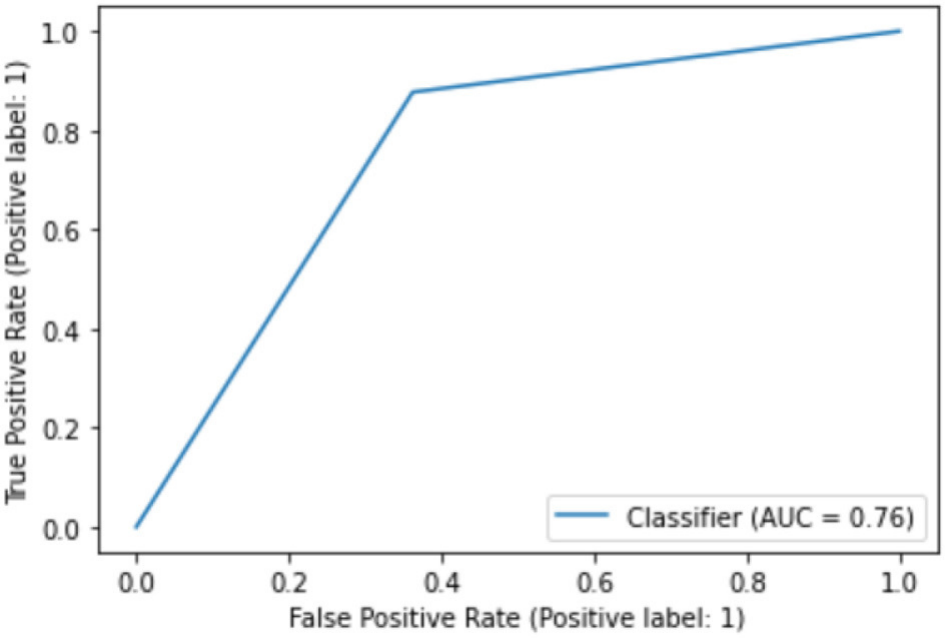
AUC on full data.

While the above performance is reported on the basis of cross validation involving both train and test data subsets for model selection and validation, a final decision tree will be trained using all available data so that data can be fully exploited in generating a working model. With an accuracy of 75.04%, this is almost the same as 75.03% as an averaged result of 10-fold cross validation. The associated AUC curve can be found in Figure 7 with more detailed results reported in Table 2. With results closely following that of Table 1, this again demonstrates the reliability of the CART model.

**Table 2:** Performance of CART model on full data.

	**Precision**	**Recall**	**F1-score**	**Support**
No (does not have ADHD)	0.85	0.64	0.73	265
Yes (has ADHD)	0.68	0.88	0.77	236
Accuracy			0.75 (overall acc: 75.04%)	501
Macro avg	0.77	0.76	0.75	501
Weighted avg	0.77	0.75	0.75	501

Furthermore, despite that the full decision tree may not be presented due to confidentiality, we are able to show the significance of variables utilized by the CART algorithm, which only includes two variables, i.e., 0.68 for with DIVA attention deficit for adulthood, and 0.32 for DIVA attention deficit for childhood, both are also the top attributes as analyzed by the correlation in Figure 6.

### 3.2. ML results without DIVAs

**Figure 8:**
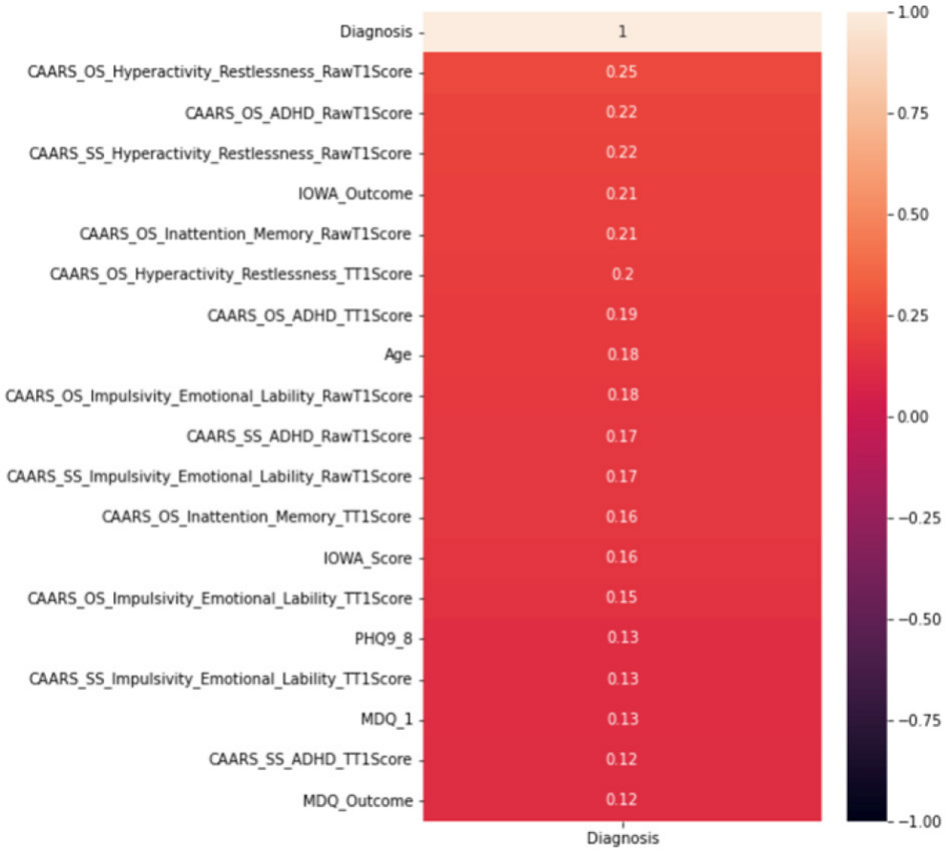
Correlation analysis of top 20 attributes excluding DIVAs.

Whilst it's assuring that the DIVA attributes exhibit strong capabilities in differentiating positive and negative ADHD cases, cost of conducting DIVA tests in practice proves high and they have to executed by senior clinicians—this motivates on-going projects to explore alternative tests that may be able to perform by junior clinicians while also being effective in the diagnosis. As such, the four attributes with DIVA tests are now removed, i.e., the DIVA Child IA and DIVA Adult IA, which is the attention deficit during childhood and adulthood; the DIVA Child HI and DIVA Adult HI. which is the Hyperactivity/impulsivity during childhood and adulthood. Similar to Figure 6, correlation of the top 20 remaining attributes with respect to the diagnosis is now shown in Figure 8, where most of the best correlated results come from the CAARS ADHD TT1 score, but the strength of correlation of these variables are much lower than that of DIVA, generally around 0.2, suggesting that it may not be valid to use each attribute alone to make effective diagnosis.

Following the same grid search of the hyper-parameters to best fit the underlying data in again 10-fold cross validation, the result of CART model is presented in Table 3, in comparison with the same set of mainstream machine learning models as above. From a holistic perspective, the averaged performance (as well as every single result) across all selected machine learning models are significantly worse than that when DIVA attributes are used in all metrics. For instance, the averaged test accuracy has dropped to 58.08% from 66.48% while the Auc is now 0.63 compared to 0.72 before. This clearly suggests how critical DIVA attributes are in establishing an effective learning model and the limited capabilities of remaining attributes. It is worth noting that both random forest and extreme gradient boosting are still able to fit the training data perfectly throughout, but their test accuracy are also very limited, suggesting that it's possible to use complex machine learning techniques like the two ensemble-based methods to fit the data, but predicting unseen ADHD patients can still be challenging, especially in the absence of DIVA attributes. Having said that, most machine learning models are still achieve results clearly better than the random guess of 52.9%.

**Table 3:** Results without DIVA.

**Data set**	**Train acc**	**Test acc**	**Balanced acc**	**Precision**	**Recall**	**F1**	**Auc**
Classification and Regression Tree	65.40	61.69	61.44	0.60	0.59	0.59	0.62
Logistic Regression	69.86	60.47	60.37	0.58	0.59	0.58	0.63
Linear Discriminant Analysis	71.55	58.66	58.60	0.56	0.57	0.56	0.60
Artificial Neural Networks	69.11	56.70	59.96	0.56	0.63	0.56	0.65
K Nearest Neighbor	75.80	59.09	59.14	0.57	0.56	0.56	0.61
Support Vector Machine (RBF)	62.74	57.71	58.84	0.60	0.49	0.51	0.66
Support Vector Machine (Linear)	72.50	59.46	59.33	0.57	0.57	0.56	0.61
Gaussian Naïve Bayes	57.35	54.26	55.66	0.51	0.81	0.62	0.61
Random Forest	100.00	58.88	59.61	0.61	0.53	0.51	0.66
Extreme Gradient Boosting	100.00	57.52	57.28	0.54	0.51	0.52	0.63
Averaged	75.43	58.08	58.75	0.57	0.59	0.55	0.63

**Figure 9:**
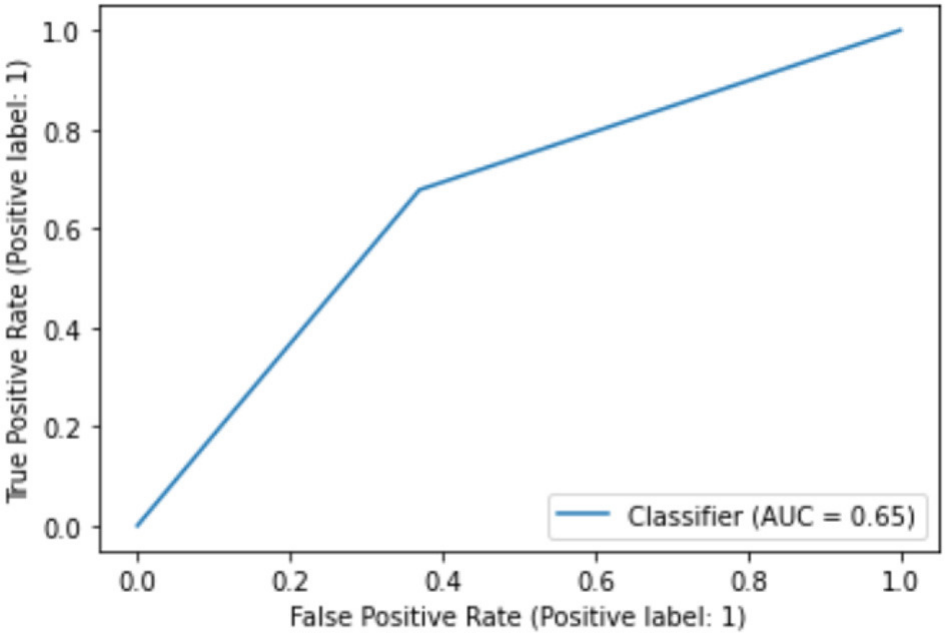
AUC on data excluding DIVA attributes.

In terms of the CART decision tree model, it still achieves the best test accuracy and balanced accuracy, with relatively small gap between train and test accuracy, indicating it still a robust and effective choice. The final decision tree model is then trained on full data again excluding the use of four DIVA attributes. The final model achieves a slightly higher accuracy of 65.27% than the averaged performance of 61.69% as a result of 10-fold cross validation. The associated AUC curve can be found in Figure 9 with more detailed results reported in Table 4. Overall, these results are slightly better than that of Table 3 obtained through the 10-fold cross validation, which can be expected as the model is trained and tested on the same data instances. As for the specific variables selected by the final CART model, irrespective of already ignored four DIVA attributes, it's observed that “IOWA_Score” is selected with 0.52 significance, followed by “Age” of 0.3 significance, and then “CAARS_OS_Inattention_Memory_TT1Score” of 0.18 importance. In comparison with the top 20 attributes in Figure 8, the three selected attributes are not the top ones as calculated by correlation, which indicates that attributes of lower correlations alone may be significant when combined with others that lead to an effective cohort.

**Table 4:** Performance of CART model on data excluding DIVA attributes.

	**Precision**	**Recall**	**F1-score**	**Support**
No (does not have ADHD)	0.69	0.63	0.66	265
Yes (has ADHD)	0.62	0.68	0.65	236
Accuracy			0.65 (overall acc: 65.27%)	501
Macro avg	0.65	0.65	0.65	501
Weighted avg	0.65	0.65	0.65	501

## 4. Results and discussions on KR and hybrid models

The knowledge model is based on if-then rules, encoding the knowledge of medical experts. In addition, the chosen machine learning algorithm, namely the decision tree algorithm, generates a set of if-then rules as well. The results of the two models are combined by the hybrid model as described in Section 2.3.3, leading to an overall prediction of an ADHD diagnosis. We evaluated all three models over the existing dataset for the 501 patients and compared the results to the diagnosis made by the medical experts. Note that the knowledge model, the machine learning model and the hybrid model are referred below as KR, ML and Hybrid models, respectively.

Table 5 shows how patients were classified by the three models. It is evident that in the ML model all patients are classified as either having ADHD or not having ADHD (Yes/No outcomes only), while in the KR model approximately 40.7% of patients are classified to a Yes/No outcome with 59.3% of patients being referred to a medical expert. It is expected that the Hybrid model will classify the minimum number of patients to a Yes/No outcome (as both KR and ML models must provide the same classification) and the maximum number of patients will be referred to a medical expert (those referred by the KR model as well as all outcome disagreements between KR and ML models). Thus, the results for the Hybrid model classifying 38.5% of patients to a Yes/No outcome and 61.5% of patients referred to a medical expert are in line with model design.

**Table 5:** Confusion matrix of KR, ML, and hybrid models.

		**Predicted**		
**Clinical outcome**	**Model**	**Yes**	**No**	**Expert**
Yes (has ADHD)	KR	57	6	173
	ML	207	29	0
	Hybrid	52	6	178
No (does not have ADHD)	KR	32	109	124
	ML	96	169	0
	Hybrid	26	109	130

Table 6 presents the accuracy of each model, namely how many patients where correctly classified out of all patients assigned to a specific set of outcomes, where the set of allowed outcomes is either Yes/No or Yes/No/Expert. Note that the highest accuracy is highlighted in bold. Referring complex cases to clinical experts increases the accuracy for both KR (from 81.37% to 92.42%) and Hybrid (from 83.42% to 93.61%) models. Recall that referring patients to medical experts is considered a valid and desirable outcome (see Section 2.3.3). The Hybrid model combines the strengths of both KR and ML models, thus exhibiting better accuracy over both Yes/No and Yes/No/Expect outcomes.

**Table 6:** Accuracy of each model per set of outcomes.

**Model**	**Yes/no (%)**	**Yes/no/expert (%)**
KR	166/204 (81.37%)	463/501 (92.42%)
ML	376/501 (75.05%)	376/501 (75.05%)
Hybrid	**161/193 (83.42%)**	**469/501 (93.61%)**

Employing Artificial Intelligence in clinical settings holds great potential to improve healthcare. However, these benefits can be attained only if the underlying ethical implications are addressed (34). Although a comprehensive ethical risk analysis is planned for future research; at this stage of this work we provide a preliminary discussion on major ethical challenges and how they are being currently addressed (when applicable) on the machine learning and knowledge-based component of the proposed framework. We cover the three primary ethical factors, as they are described in (35), namely, data protection, algorithmic fairness and accountability.

The dataset used to train the machine learning component was provided by SWYPFT following Caldicott Principles (Section 2.1) and privacy is ensured via anonymization, where individuals are no longer identifiable. Clinical data are primarily used to train the machine learning component by recognizing data patterns and encoding them into the underlying mathematical formulation of the model. The knowledge-based part, on the other hand, does not rely on data but expert knowledge. Data protection is assured considering that drawing predictions using the trained model or the rules within the knowledge-based model does not provide any access to the initial dataset nor the data of a new case is internally stored.

In terms of algorithmic fairness, the proposed framework incorporates several steps to reduce bias. The training dataset is relatively balanced and includes all the available assessments within the predefined case study period, making the dataset representative of the selected demographic. Several candidate models are trained using cross-validation to mitigate bias by minimizing the odds of over-fitting. This is a crucial step that prevents models from learning particularities of the training dataset and instead enables them to focus on more generic data trends. In the case of hyperparameters, several performance metrics are employed and tuning is achieved via a thorough grid search.

Regarding accountability, the proposed work operates as a recommendation system that aims to assist clinicians instead of independently ruling diagnostic decisions. Even as a decision support tool, explainability plays a pivotal role when applying AI in clinical settings. Consequently, great emphasis is put on transparency, where both knowledge-based model (rule-based format) and the optimal machine learning model (CART—decision tree structure) provide clear reasoning paths that facilitate *in situ* examination of potential outcomes.

Several ethical considerations have been taken into account and the corresponding ethical issues have been mitigated through sophisticated design of the tasks of data processing, training pipeline and knowledge representation. However, the resources that build the proposed framework, clinical data and expert medical knowledge are of high importance and they may raise ethical challenges if the quality is not assured, despite the rigorous design of the methodology. For instance, expert systems that partially capture the available knowledge (e.g., ignoring special cases—outliers—of ADHD) or non-representative clinical data (due to data scarcity) can introduce bias in the end product. In the current work, the models rely on carefully curated information provided by the collaborating healthcare facility that meet quality standards to examine the capabilities of the proposed solution. We plan to advance this work to a wider clinical study and eventually pilot this solution into a fully-fledged AI diagnostic recommendation tool. However, this would require an in-depth analysis that would eliminate any bias, which is one of the first priorities of our future work.

## 5. Conclusion

This paper is part of our long-term effort to introduce automation support to the diagnosis of adult ADHD, using AI technologies. Following clinical trial deployment in an NHS adult ADHD service, this paper reported on results obtained from retraining machine learning models on the richer dataset. The results are encouraging and suggest that the AI algorithm can be used in clinical practice.

Next steps in our efforts will include obtaining a broader evidence base by trialing the AI algorithm in other NHS or private healthcare providers. In addition, performing an in-depth ethical risk analysis and introducing mitigation strategies to eliminate bias is included in our project plan. Furthermore, we will validate and refine the knowledge-based model to confirm it captures all relevant knowledge and introduce flexibility in the contained rules using exceptions of probabilities. We also intend to conduct a study on the acceptance of our approach by clinicians and patients.

We also trained machine learning models *without using DIVA attributes*, so as to potentially lower the burden of the diagnostic process on healthcare services. The accuracy obtained was not sufficiently high to encourage clinical testing. We believe that this outcome is partially due to the absence of a knowledge model that could work in conjunction with the machine learning model—note that the knowledge model of (19) could not be used because it makes uses of DIVA values. In future work, we intend to develop a new knowledge model without use of DIVA attributes, to help increase overall accuracy through a hybrid AI algorithm.

## Data availability statement

The datasets presented in this article are not readily available because in the interests of protecting patients' privacy, the data cannot be shared. Requests to access the datasets should be directed to MA, marios.adamou@swyt.nhs.uk.

## Ethics statement

Ethical approval was waived by South West Yorkshire Partnership Foundation Trust (SWYPFT) Research and Development Department as data were gathered retrospectively. Written informed consent was not required in accordance with institutional requirements and national legislation.

## Author contributions

TC: formal analysis, literature research, methodology, validation, and writing—draft and review. IT: formal analysis, methodology, validation, and writing—draft and review. SB and EP: writing—review and editing. MA: conceptualization and methodology. GA: conceptualization, methodology, and writing—review and editing. All authors contributed to the article and approved the submitted version.
